# Supplementary material for: Chromothripsis during telomere crisis is independent of NHEJ, and consistent with a replicative origin
Source: Genome Res. 2019 May;29(5):737–49. doi: 10.1101/gr.240705.118 (PMC6499312; doi:10.1101/gr.240705.118)
Supplement: Supplemental Material [file supp_gr.240705.118_Supplemental_file_1.zip › contigs/annotated_contigs/DB82/contig.2.DB82_length_350_mean_cov_8.08571428571.docx]

**DB82_length_350_mean_cov_8.08571428571**

```
CTCACTCTGTCGCCCAGGCTGGAGTGCAGTGGTGCAATGAAGGCTCACTGTAACCTCTGCTTCCTGGGTTCAAATGATTCTCCTGCGCC
 >chr22:38892380-38892504 + E=3e-60
TCAGCCTCCTGAGTAGCTGGGACTAGAGGCAGGCA|TGCCTCTAGT|GTGTGGCGGCGCATGCCTGTAATCCCAGCTGCTTGGGAAGCT
                                              >chr22:38893159-38893380 + E=5e-109
GAGACAGGAGAATCGCTTGAAGCCAGGAGGTGGAGGTTGCAATGAGCCGAGATTGTGCCAATGTACTCCAGCGTGGGCAACCAAGAGCG

AAACTCCATCTCAAAAAAAAAAAAAAAAAAAAGTACACATTCAATGCCAGGCGCGGTGGCTCACACCTGTAATCCCAGCACCTTG
```
